# Fabrication of an Eco-Friendly Clay-Based Coating for Enhancing Flame Retardant and Mechanical Properties of Cotton Fabrics via LbL Assembly

**DOI:** 10.3390/polym14224994

**Published:** 2022-11-18

**Authors:** Mingjia Kang, Silu Chen, Rongjie Yang, Dinghua Li, Wenchao Zhang

**Affiliations:** National Engineering Research Center of Flame Retardant Materials, School of Materials Science and Engineering, Beijing Institute of Technology, Beijing 100081, China

**Keywords:** intumescent flame retardants, clay-based materials, cotton, layer by layer assembly, mechanical property

## Abstract

An eco-friendly clay-based synergistic flame-retardant coating was established on cotton fabrics via facile layer-by-layer assembly derived from polyethyleneimine (PEI), attapulgite clay (ATP), and phytic acid (PA). The fabricated flame-retardant (FR) cotton fabrics demonstrated improved thermal stability. Compared to untreated cotton fabrics, the limiting oxygen index of Cotton-8TL was improved to 27.0%. The peak heat release rates of the prepared FR cotton fabrics were lower than that of the pristine cotton fabrics, showing a maximum reduction of 41%. The deposition coating system improved the amount of char residue effectively. The intumescent flame-retardant mechanism was proposed through the analysis of char residue and the suppression properties of volatile gases. Furthermore, compared with those of the untreated cotton fabrics, the tensile strength and elongation at break of the FR cotton fabrics in the warp direction were improved by 20% and 47% remarkably, respectively. A feasible surface modification strategy was provided for the flame-retardant treatment of cotton fabrics with the improvement of mechanical properties.

## 1. Introduction

Cotton is one of the widely used traditional cellulose materials which occupies a large share of the textile market. Due to its advantages such as natural abundance, comfortableness, softness, hydrophilicity, air permeability, and biodegradation, cotton fabrics are widely used in residential, industrial, medical, and other fields [1,2]. However, the flammability of cotton fabrics in the air poses a serious challenge to its practical application. Therefore, the flame-retardant treatment of cotton fabrics is an integral part of the production.

The main component of cotton is cellulose. Abundant hydroxyl groups of cellulose allow extensive surface modification through chemical reactions, the formation of both intermolecular and intramolecular hydrogen bonding [3,4], among others. With the rapid development of flame retardant technology, many methods are applied to flame retardant treatment for textiles including dip coating [5,6,7], spray coating [8], chemical grafting [9,10], plasma treatment [11,12], sol-gel method [13,14,15], and layer-by-layer (LbL) assembly [16,17,18]. The LbL assembly method is a versatile and cost-effective approach to fabricating multilayers on various substrates involving foams, fabrics, paper, and bulk polymers [19]. The coating layers were fabricated on the surface of substrates based on the alternating deposition of solutions with instinct properties. The thickness of the coating is controllable on a nanoscale through changing the coating layer numbers. Besides traditional electrostatic interaction, hydrogen bonding, covalent bonding, and donor/acceptor interaction can be employed so that the range of suitable materials which can be used in the LbL assembly is expanded including biomolecules, and organic and inorganic nanoparticles [20,21]. Hence, the LbL assembly method shows a promising technique for flame-retardant surface treatment.

**Figure 1:**
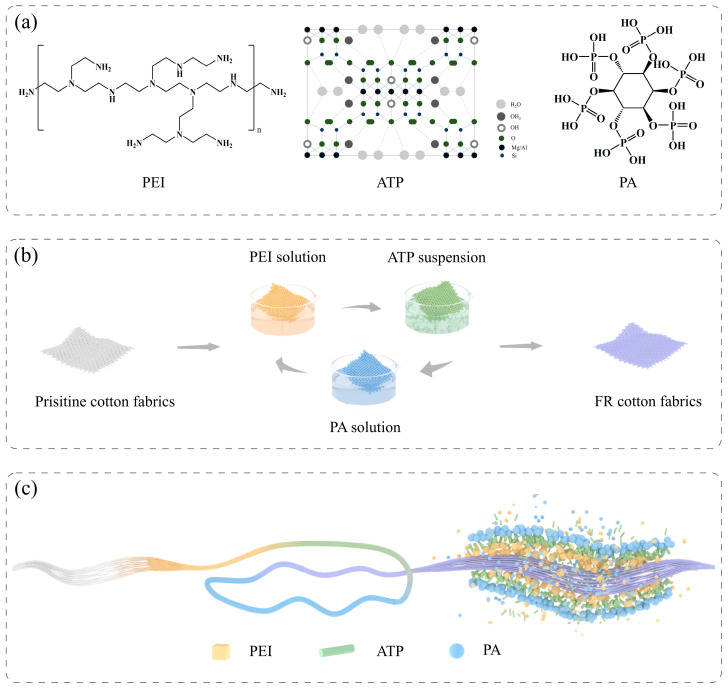
(**a**) Chemical structure of PEI and PA and the crystal structure of ATP, (**b**) diagrammatic illustration of LbL assembly process, and (**c**) the change of a single cotton fiber during the LbL treatment.

In recent years, eco-friendly flame retardants were explored to replace halogen-containing flame retardants due to being toxic and smoky, and other drawbacks [22]. Phosphorus (P), nitrogen (N), and silicon (Si) containing flame retardants are regarded as promising halogen-free alternatives [23,24,25,26]. Bio-based materials have already attracted attention to producing eco-friendly flame retardants including chitosan (CH) [27,28,29], deoxyribonucleic acid (DNA) [30,31,32], phytic acid (PA) [33,34], polydopamine (PDA) [35], starch [36], cyclodextrin [37,38], lignin [39], and tannic acid (TA) [40,41]. Due to the diversity of the materials for the LbL assembly, intumescent flame retardants which show high efficiency, low smoke, and low toxicity were developed through this method recently. Typical intumescent flame retardants are composed of an acid source, a carbon source, and a blowing agent [42]. PEI is a favorable blowing agent which is utilized to develop intumescent flame retardant systems (Figure 1a) [43]. Li et al. established a flame-retardant coating with branched PEI and sodium montmorillonite (MMT) and studied the effects of the pH value of branched PEI on flame retardancy. The FR cotton fabrics with a pH value of 10 show a greater mass gain, which might be attributed to the differences in the adhesion between fabrics and polyelectrolytes [44]. Owing to the high P content (28%), PA was used widely as a P-containing flame retardant (Figure 1a). During the degradation of cotton fabrics, PA can act as a catalyst to promote char formation [45]. Zhang et al. adopted CH and PA to form an intumescent flame-retardant system with metal ions as a synergist on cotton fabrics. After the treatment, the peak heat release rate (PHRR) of CH/PA/Ba/PA was reduced by 61% compared with that of uncoated cotton fabrics [28].

Not only the flame retardancy but also other properties of the cotton fabrics need to be taken into consideration during the surface treatment process. The deterioration of the mechanical property usually happens after flame-retardant treatment. Zhang et al. developed a green flame-retardant coating on cotton fabrics using TA, tartar emetic, and Fe^2+^. The tensile strength in the warp direction decreased from 37.30 MPa to 25.33 MPa due to the damage of TA to the fibers [41]. Thi et al. prepared the FR cotton fabrics treated with plasma to activate the surface before flame-retardant treatment [12]. Although the FR cotton fabrics with pre-activation exhibited good flame retardancy, the mechanical strength show a 30–50% loss, resulting from inevitable structure damage by the plasma treatment. Therefore, balancing flame retardancy with mechanical properties is necessary. As shown in Figure 1a, attapulgite clay (ATP) is a silicate mineral that contains flame-retardant elements including Si, magnesium (Mg), and aluminum (Al) [46]. ATP has high thermal stability, a large specific surface area, and excellent hydrophilicity. Bao et al. grafted ATP on the surface of cotton fabrics through a thiol-ene click reaction. The limiting oxygen index (LOI) value of the treated cotton fabrics was 25.6%. In addition, the presence of ATP improved tensile strength at the break by 10.59% [47]. Gao et al. synthesized the poly(acrylic acid)/attapulgite (PAA/ATP) nanocomposites to treat cotton fabrics in which the LOI value increased to 22.7%. Compared with the FR cotton fabrics treated only with PAA, the breaking strength after the surface treatment with PAA/ATP nanocomposites was improved because of the ester bond crosslinking [48]. Instead of chemical modification, LbL assembly can offer a facile approach to comprehensive improvement of flame retardancy and mechanical properties.

This research work aims at endowing the cotton fabric with both good flame retardancy and favorable mechanical properties. An effective synergistic flame-retardant system was developed by LbL assembly. The synergistic flame-retardant coatings consisted of an intumescent flame-retardant system and inorganic mineral clay. Specifically, branched PEI, PA, and cotton were used as the blowing agent, acid source, and carbon source, respectively. ATP was employed to play a synergistic role in the system. The morphology and chemical composition of FR cotton fabrics before and after combustion were analyzed. The effects of the coating layer number on the thermal stability and flame retardancy of cotton fabrics were investigated. Besides, the mechanism of the flame retardant was proposed based on the analysis of gaseous and condensed phases. The mechanical properties of FR cotton fabrics were studied, and the morphology and structure of the fracture areas were also analyzed.

## 2. Materials and Methods

### 2.1. Materials

Cotton fabrics (100%, 135 g/m^2^) were supplied by Hongda Fabric Industry (Hebei, China). Polyethyleneimine (PEI, branched, Mw = 10,000) was purchased from Saan Chemical Technology Co., Ltd. (Shanghai, China). Phytic acid (PA, 70 wt% aqueous solution) was purchased from Aladdin Chemistry Co., Ltd. (Shanghai, China) Attapulgite (ATP) was purchased from Shanghai Yuanye Bio-Technology Co., Ltd. (Shanghai, China). Sodium hydroxide (NaOH) and hydrochloric acid (HCl) were purchased from Beijing Tongguang Fine Chemicals Co., Ltd. (Beijing, China). Deionized water was used as solvent throughout the experiments.

### 2.2. Preparation of Acid-Treated ATP

The original ATP was treated with HCl to activate its surface. First, 25 g of ATP was added to the HCl solution (250 mL, 1 mol/L), and the mixture was stirred vigorously at 50 °C for 1 h. The suspension was then centrifuged at 5000× *g* rpm for 5 min and dried at 60 °C for 24 h to obtain the acid-treated ATP.

### 2.3. Preparation of FR Cotton Fabrics

1 wt% PEI solution was obtained by dissolving PEI into deionized water, and the pH value of the solution was adjusted to 9.5 using 1 mol/L of HCl solution. The pH value of 1 wt% PA solution was adjusted to 4 using 1 mol/L of NaOH solution. The 0.6 wt% ATP suspension was initially sonicated for 10 min by an ultrasonic cell disruptor for better dispersion, and the pH value was adjusted to 5 using 1 mol/L NaOH solution. All the cotton fabrics were pretreated with deionized water and alkaline solution to remove the impurities. As shown in Figure 1b, pretreated cotton fabrics were alternately dipped into the prepared PEI solution, ATP suspension, and PA solution in sequence so that one trilayer (TL) was established. The immersion time was 10 min for the first round and 3 min for later rounds. The extra solution or suspension was removed by pressing and squeezing during the deposition process. When the coating number achieved 2, 4, and 8, respectively, the FR cotton fabrics (designated as Cotton-2TL, Cotton-4TL, and Cotton-8TL) were then dried at 60 °C for 1 h. The cotton fabrics after pretreatment (designated as Control) were used as control samples.

### 2.4. Characterizations of the Control and FR Cotton Fabrics

The morphology of the samples was observed by scanning electron microscopy (SEM, Hitachi S4800, Tokyo, Japan) at 3 kV equipped with an energy dispersive spectrometer (EDS). The chemical structure and composition of the samples were characterized by Fourier transform infrared in the frequency region of 4000–400 cm^−1^ (FTIR, Thermo Scientific Nicolet iS10, MA, USA), X-ray photoelectron spectroscopy (XPS, Ulvac-Phi PHI Quantera II, Chigasaki, Japan) equipped with standard monochromatic Al-Ka radiation (ht = 1486.6 eV), and X-ray diffraction (XRD, Rigaku MiniFlex 600, Tokyo, Japan) at 36 kV and 20 mA. Raman spectra were obtained from a Laser Raman spectrometer (Renishaw inVia Qontor, Gloucestershire, UK) in the range from 800 to 2000 cm^−1^ using the 514 nm argon laser line. The mechanical properties of samples were measured using a microcomputer-controlled electronic universal testing machine (MTS Systems Corporation CMT 4104, Eden Prairie, MN, USA) according to the standard GB/T 3923.1-2013.

The thermogravimetric analysis (TGA) was carried out on a thermogravimeter (Mettler Toledo TGA/DSC3+, Zurich, Switzerland) under a nitrogen (N_2_) atmosphere from 30 to 700 °C at a linear heating rate of 10 °C/min, with a N_2_ flow rate of 50 mL/min. LOI test was performed on an intelligent critical oxygen index analyzer (Testech TTech-GBT2406-1, Suzhou, China) based on the standard ISO 4589-2:2017. The combustion test was performed using the cone calorimeter (Fire Testing Technology, East Grinstead, UK) according to the standard ISO 5660-1:2015. Each sample with a dimension of 100 mm × 100 mm was exposed to the external heat flux of 35 kW/m^2^. The vertical flame test (VFT) was performed on an automatic vertical flammability cabinet (Nanjing Jiangning Analysis Instrument Co., Ltd. (CZF-3, Nanjing, China) according to GB/T 5455-2014. Thermogravimetric analysis-infrared spectrometry (TG-IR) was obtained by a thermogravimeter (Mettler Toledo TGA/DSC3+, Zurich, Switzerland) combined with a FTIR spectrophotometer (Thermo Scientific Nicolet iS50, Waltham, MA, USA). The samples were heated under N_2_ atmosphere from 30 to 700 °C at a heating rate of 10 °C/min.

## 3. Results and Discussion

### 3.1. Characterizations of the Control and FR Cotton Fabrics

FR cotton fabrics were obtained via LbL assembly with polycationic electrolytes (PEI), inorganic particles (ATP), and anionic species (PA). As shown in Figure 1b, untreated cotton fabrics were immersed in PEI solution, ATP suspension, and PA solution alternatively. Besides the strong electrostatic interaction between PEI and PA, numerous amino groups on PEI could form hydrogen bonds with the hydroxyl groups on the surface of cotton fabrics and ATP. Therefore, PEI acted as a binder between cotton fabrics and other species [43]. PEI and PA could be attached to ATP due to van der Waals forces. Furthermore, PEI, ATP, and PA were not only coated on the surface but also infiltrated the interior of the fibers during the LbL process as shown in Figure 1c, which could impact the flame retardancy and mechanical properties of the prepared cotton fabrics.

**Figure 2:**
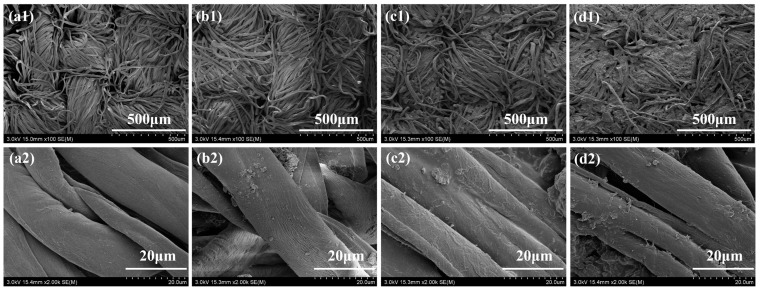
SEM images of all cotton fabrics: (**a1**,**a2**) Control, (**b1**,**b2**) Cotton-2TL, (**c1**,**c2**) Cotton-5TL, (**d1**,**d2**) Cotton-8TL.

The SEM images were utilized to investigate the morphology of the control and FR cotton fabrics. As shown in Figure 2, the surface of the control cotton fabrics was very smooth, while a coating layer appeared on the surface of the fibers after the LbL assembly. Remarkable wrinkles and bumps were observed which were derived from the aggregation of clay particles owing to the interactions with electrolytes. The mass gain was calculated by the following equation:

(1) $$add\text{-}on=\frac{m_{1}-m_{0}}{m_{0}}\times 100\%$$

where *m*_0_ and *m*_1_ represent the mass of the cotton fabrics before and after the LbL treatment, respectively. The add-ons of the Cotton-2TL, Cotton-5TL, and Cotton-8TL were 10.5%, 28.3%, and 32.7%, which increased gradually. Hence, the gaps between the fibers created by the weaving process became less visible.

XPS was employed to investigate the element composition on the surface of the control and FR cotton fabrics (Figure S1). Compared with the control samples, new N 1s, P 2p, and Si 2p signals appeared in the wide-scan XPS spectra of FR cotton fabrics. In Table 1, the atomic concentration of carbon (C) decreased as the number of coating layers increased, while the atomic concentration of N increased, which indicated that more PEI was deposited on the surface of cotton fabrics. Additionally, the elements silicon, magnesium, and aluminum only exist in ATP, and the sum atomic percentages of these three atoms in fabricated FR cotton fabrics were 2.75%, 4.43%, and 4.72%, respectively. Since there were van der Waals forces and hydrogen bonds between ATP and polyelectrolytes, the three materials were deposited on the surface of the cotton fabrics alternatively as described in Figure 1c. In addition, the elemental distribution of Cotton-8TL fabrics was observed by EDS (Figure S2). Both the results of XPS and EDS confirmed that the flame-retardant coating layers were deposited successfully.

The chemical composition of the control and FR cotton fabrics was characterized by FTIR (Figure S3). The absorbance bands at around 3306 cm^−1^ were attributed to the stretching vibration of -OH in cellulose. The absorption peaks at 2901 cm^−1^ were assigned to the stretching vibration of the aliphatic C-H bond. The peaks at 1159 cm^−1^, 1109 cm^−1^, and 1028 cm^−1^ belonged to the C-O-C and C-O groups that existed in the cellulose backbone. The peaks at 1439 cm^−1^ and 1315 cm^−1^ were assigned to the bending vibration of aliphatic -CH_2_- and -CH-, respectively. The overlapping peak at around 1631 cm^−1^ was assigned to the bending vibration of the N-H bond in PEI and the -OH bond in ATP and PA. Owing to more deposition of the flame-retardant coating layers, the peak at 1631 cm^−1^ became much more obvious. A new absorption peak at 784 cm^−1^ was observed in the FR cotton fabrics, which corresponded to the stretching vibration of Al-O-Si in ATP. Hence, there was no new chemical bond forming during the LbL assembly process. The deposition between coating layers predominately relied on electrostatic interaction, hydrogen bonding, and other intermolecular forces.

### 3.2. Thermal Stability of the Control and FR Cotton Fabrics

**Figure 3:**
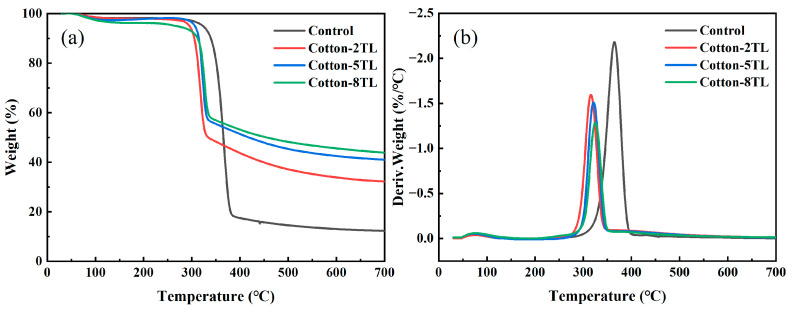
(**a**) TGA and (**b**) DTG curves of the control and FR cotton fabrics.

TGA provides the thermal degradation of the control and FR cotton fabrics by measuring their mass loss as a function of temperature. The TGA and DTG curves tested in the N_2_ atmosphere are shown in Figure 3, and all the samples had a similar thermal degradation trend. T_-5%_ and T_max_ represent the 5 wt% weight loss temperature (initial degradation temperature) and the maximum degradation temperature, respectively. The T_-5%_ of the control cotton fabrics was 323.3 °C, while the FR cotton fabrics possessed a lower temperature (Table S1). The T_-5%_ of Cotton-8TL decreased to 268.5 °C due to the early degradation of PEI and PA. The decomposition of PA produced acid compounds which were regarded as catalysts and promoted the dehydration reaction and carbonization of cotton fabrics. As a result, the char yield at 700 °C was improved with the increase of coating layers. The char residue of Cotton-8TL was much more than three-fold that of the control cotton fabrics. ATP can convert into a series of inorganic oxides during the combustion process [48]. Therefore, ATP took part in the improvement of thermal stability and char residue. Not only the T_max_ but also the maximum mass loss rate of the FR cotton fabrics decreased in comparison to those of the control cotton fabrics. Earlier decomposition of flame retardants promoted char formation which could block mass transfer and further hinder the degradation of inner cotton fabrics.

### 3.3. Flame Retardancy of the Control and FR Cotton Fabrics

**Figure 4:**
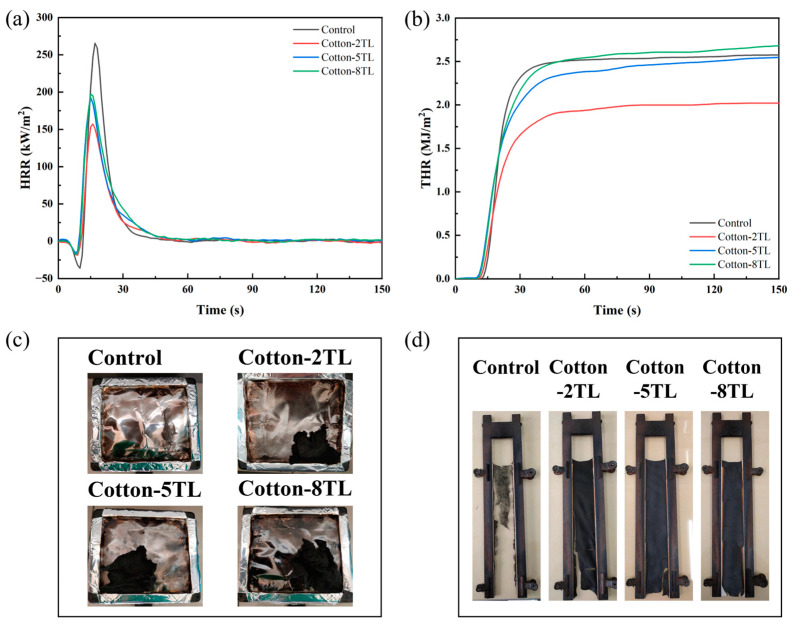
(**a**) HRR curves, (**b**) THR curves, and digital photos of the control and FR cotton fabrics after (**c**) CCT and (**d**) VFT.

In general, pristine cotton fabrics have a low LOI value which needs to be improved. The effect of coating layer number on the LOI values was studied. As exhibited in Table 2, the control cotton fabrics were so flammable that the LOI value was only 17.2%. After the LbL assembly treatment, the LOI values of the FR cotton fabrics were improved effectively. The LOI values of FR cotton fabrics increased as the number of coating layers increased. Among them, the LOI value of the Cotton-8TL increased by 57% compared with that of the control cotton fabrics. Therefore, this implies that the increase of the coating layers can improve the LOI value of the cotton fabrics. VFT was used to evaluate the flame retardancy of the prepared FR cotton fabrics as well. The untreated cotton fabrics were ignited immediately and burned completely with 14.6 s for afterflame time and 31.7 s for afterglow time. The FR cotton fabrics had a reduction of 2–6 s for afterflame time, and there was no afterglow found once the flame on the fabrics disappeared; all the FR cotton fabrics apparently left more char residue after burning than the control cotton fabrics as shown in Figure 4d.

CCT is one of the most effective fire behavior measurements and can gather quite a few parameters associated with the burning properties of the samples. The PHRR, total heat release (THR), and the fire growth rate (FIGRA) index of the control and the FR cotton fabrics are described in Table 2, and the variation of heat release rate (HRR) and THR with time are shown in Figure 4a,b. With the help of the flame-retardant treatment, all the FR cotton fabrics had lower PHRR values than the control cotton fabrics. Most obviously, the PHRR value of the Cotton-2TL was reduced by 41% which was the lowest PHRR value of the FR cotton fabrics (157.2 kW/m^2^). However, the PHRR values tended to increase with further increase of the coating layers. The variation trend of THR was consistent with that of PHRR, and the THR value of Cotton-2TL was decreased by 22% compared to that of the control cotton fabrics. Thicker coating layers could result in more aggregation of the flame-retardant components during the combustion process, which was confirmed in the analysis of the char residue [49]. It might lead to the appearance of some cracks in char residue [50,51]. Besides, the bubbles of the intumescent flame-retardant coatings distributed on the fabric surface appeared to be smaller as more layers were constructed, leading to a dense char layer. Therefore, increasing coating layers could not always achieve low heat release. The FIRGA index is an essential parameter to evaluate both fire hazard and predicted fire spread rate and can be calculated by dividing the PHRR by the time to PHRR [18]. The FIRGA index of the control cotton fabrics was as high as 15.6 kW/m^2^/s, whereas those of FR cotton fabrics diminished, which implies the fire hazards were suppressed effectively.

### 3.4. Analysis of Char Residue of the Control and FR Cotton Fabrics

**Figure 5:**
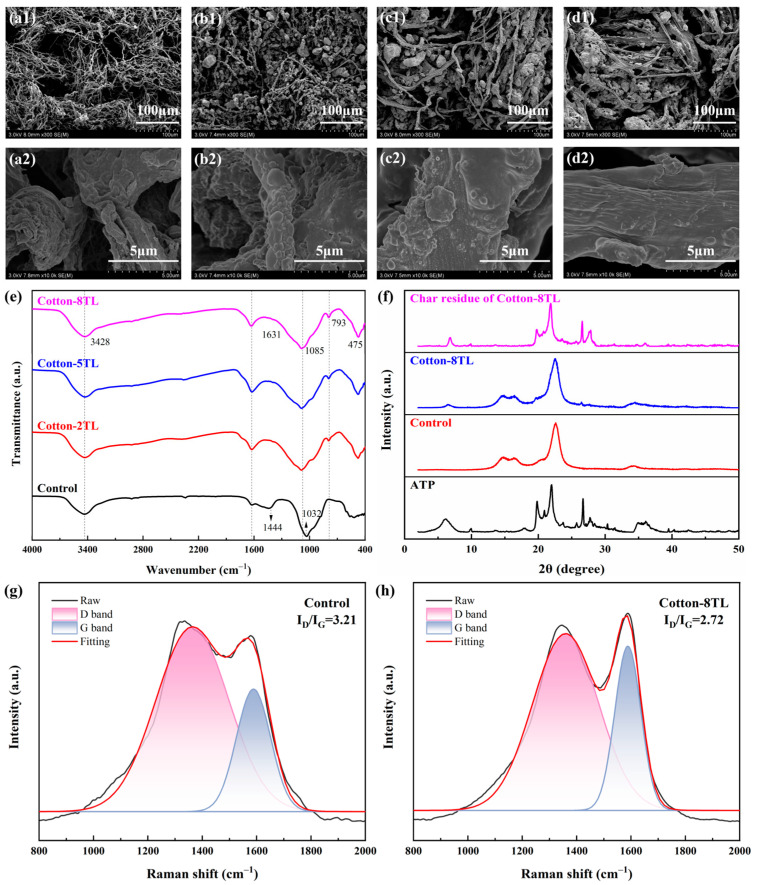
SEM images of (**a1**,**a2**) Control, (**b1**,**b2**) Cotton-2TL, (**c1**,**c2**) Cotton-5TL, (**d1**,**d2**) Cotton-8TL after CCT in different magnifications. (**e**) FTIR spectra of the char residue for the control and FR cotton fabrics after CCT. (**f**) XRD patterns of ATP, Control, Cotton-8TL, and Cotton-8TL char residue. Raman curves of char residue of (**g**) Control and (**h**) Cotton-8TL.

The digital photos of the char residue were taken after CCT, and it is observed that the control cotton fabrics were pyrolyzed completely and almost no char residue remained after combustion (Figure 4c). On the contrary, the FR cotton fabrics left more char residue, indicating less fuel transferred to the external flame zone. As shown in Figure 5a–d, although the woven structure of the control cotton fabric’s char was retained, the structure of the inner fibers was destroyed completely, while intumescent char layers of the FR cotton fabrics formed. During the combustion process, some inert gases were released but trapped inside the melting char, resulting in bubbles on the surface of the FR cotton fibers. Furthermore, a large amount of lumpy aggregation was interspersed among the fibers and was mainly composed of the products of ATP after CCT (Figure S4). As the layer numbers increased, the volume of the lumpy aggregation became larger, which was mainly attributed to more deposition of ATP due to the van der Waals forces and hydrogen bonds between ATP and electrolytes. As the number of coating layers increased, the char residue on the surface of the FR cotton fabrics became much denser. Therefore, the bubbles on the surface of the char residue were smaller with increasing layer numbers.

The FTIR spectra of the char residue for the control and Cotton-8TL after CCT were analyzed. As shown in the spectrum of the char for the control cotton fabrics in Figure 5e, the absorption peak at 1444 cm^−1^ was assigned to the aromatic C=C stretching vibration. The peak at 1032 cm^−1^ was attributed to the C-O-C bond, which revealed the formation of ether in the char residue. In the spectrum of Cotton-8TL char residue, the peak at 1631 cm^−1^ was clear, implying the overlapping vibration areas of the N–H bond in amine, -OH bond in phosphorus compounds, and the aromatic C=C bond. The peaks at 1085 cm^−1^ indicated that ether compounds were produced in the char of the FR cotton fabrics. Besides, the absorption peaks at 793 and 475 cm^−1^ corresponded to the Al-O-Si bond and P-O bond. The existence of the Al-O-Si bond in the char residue of Cotton-8TL indicated the presence of ATP residue which could achieve flame retardancy by forming a dense physical barrier.

The structural alterations of the control and Cotton-8TL before and after CCT were characterized by XRD. As depicted in Figure 5f, the control cotton fabrics displayed the typical crystal structure with three broad peaks at 14.6°, 16.3°, and 22.6°, and the characteristic peaks of ATP were at 6.1°, 19.8°, 22.0°, and 26.7° [48]. After the LbL assembly, a new peak appeared at 6.4°, which implied that the deposited ATP on the surface of FR cotton fabrics existed. Prolonged exposure to 35 kW/m^2^ heat flow brought about the breaking of hydrogen bonds, destruction of the chemical structure during the initial pyrolysis process, and the subsequent aromatization in the char residue [52]. These processes resulted in the loss of the crystal structure and the formation of the highly disordered structure in cellulose char. Therefore, all the characteristic peaks that appeared at the pattern of Cotton-8TL after combustion belonged to the oxide isolation layer produced from ATP which were mainly SiO_2_, MgO, and Al_2_O_3_. The oxide isolation layer was of good thermal stability, which acted as a shield to retard the mass and heat transfer.

Raman spectra of the CCT char residue for the Control and Cotton-8TL were further adopted to characterize the graphitization degree. As shown in Figure 5g,h, there were two peaks at around 1360 cm^−1^ and 1590 cm^−1^ which is the so-called D and G band, respectively [53]. The D band is attributed to a hybridized vibrational mode associated with the disorder of the graphene structure, and the G band originates from the E_2g_ stretching vibration mode of carbon pairs in the aromatic ring [41,54]. The intensity ratio of the D band and G band (I_D_/I_G_) was utilized to evaluate the graphitization degree. The I_D_/I_G_ value of the control cotton fabric’s char residue was 3.21, while the char residue of the Cotton-8TL shows a lower I_D_/I_G_ value of 2.72. This demonstrates that stable and dense graphitized char residue of the FR cotton fabrics could be formed under a high temperature.

### 3.5. Suppression Properties of Volatile Gases

**Figure 6:**
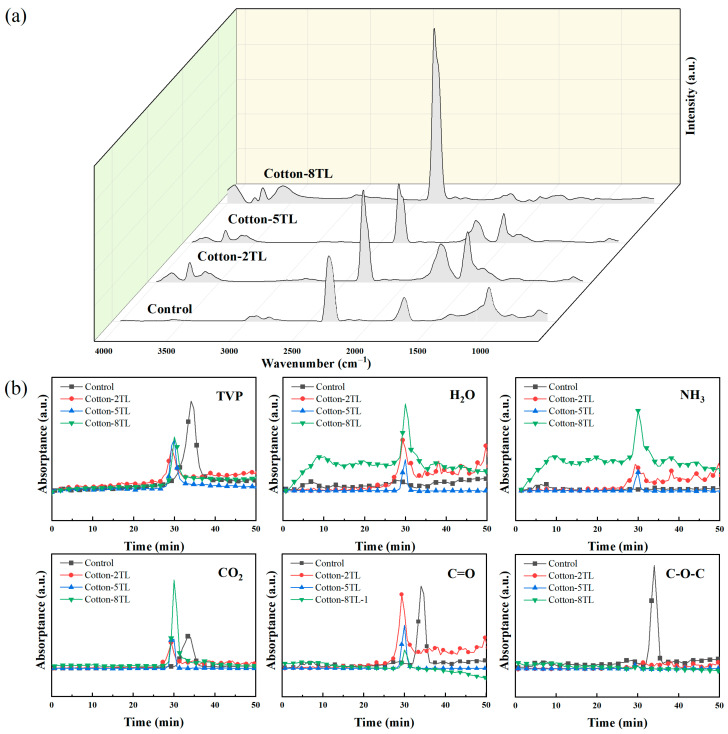
(**a**) FTIR spectra at T_max_ and (**b**) FTIR absorbance of decomposed volatiles of the control and FR cotton fabrics.

The FTIR spectra of products from the pyrolysis of the control and FR cotton fabrics were obtained by TG-IR tests. FTIR spectra at T_max_ of the samples are shown in Figure 6a; the peaks at 3587 cm^−1^ were assigned to the -OH groups of released water. The absorption peak at 2819 cm^−1^ was assigned to the C-H bond derived from the aliphatic compounds. The peak at 2345 cm^−1^ was attributed to the stretching vibration of CO_2._ The peak at 1743 cm^−1^ was assigned to C=O bonds. The absorption peak that appeared at 1068 cm^−1^ was mainly ascribed to the C-O-C bonds of ether. Concerning the spectra of the FR cotton fabrics, bonds at about 3730 cm^−1^ were attributed to the -OH in H_2_O. The absorption peaks at around 3606 cm^−1^ and 1520 cm^−1^ were attributed to the stretching and bending vibration of N-H groups, respectively, which revealed that NH_3_ was yielded during the pyrolysis process_._ Additionally, the FTIR spectra of the total pyrolysis products and specific groups including H_2_O, NH_3_, CO_2_, C=O, and C-O-C versus time are shown in Figure 6b. The release peak of the pyrolysis products for the FR cotton fabrics appeared 14 min earlier than that of the control owing to the catalytic decomposition effect of the coatings [55]. It was consistent with the thermal degradation in the results of the TGA test. Compared with the control cotton fabrics, the absorption peak of C-O-C disappeared in the spectra of the FR cotton fabrics, which implied that flammable ether did not generate. The C=O absorption intensities of all FR cotton fabrics were lower than that of the control cotton fabrics, indicating a smaller amount of flammable gas was produced. Furthermore, the absorption intensity decreased with the increase of coating layers. There were more nonflammable gases released for the FR cotton fabrics, and the absorption intensities of H_2_O and CO_2_ were much higher than those of the control. ATP contains free and bound water which was released during the process. Besides, another inert gas, NH_3_, was produced during the pyrolysis process. As the layer numbers increased, the absorption intensities of H_2_O, CO_2,_ and NH_3_ increased. All nonflammable gases could dilute the concentration of O_2_ and flammable gases in the combustion process and obstruct the fuel supply.

### 3.6. Flame-Retardant Mechanism

**Figure 7:**
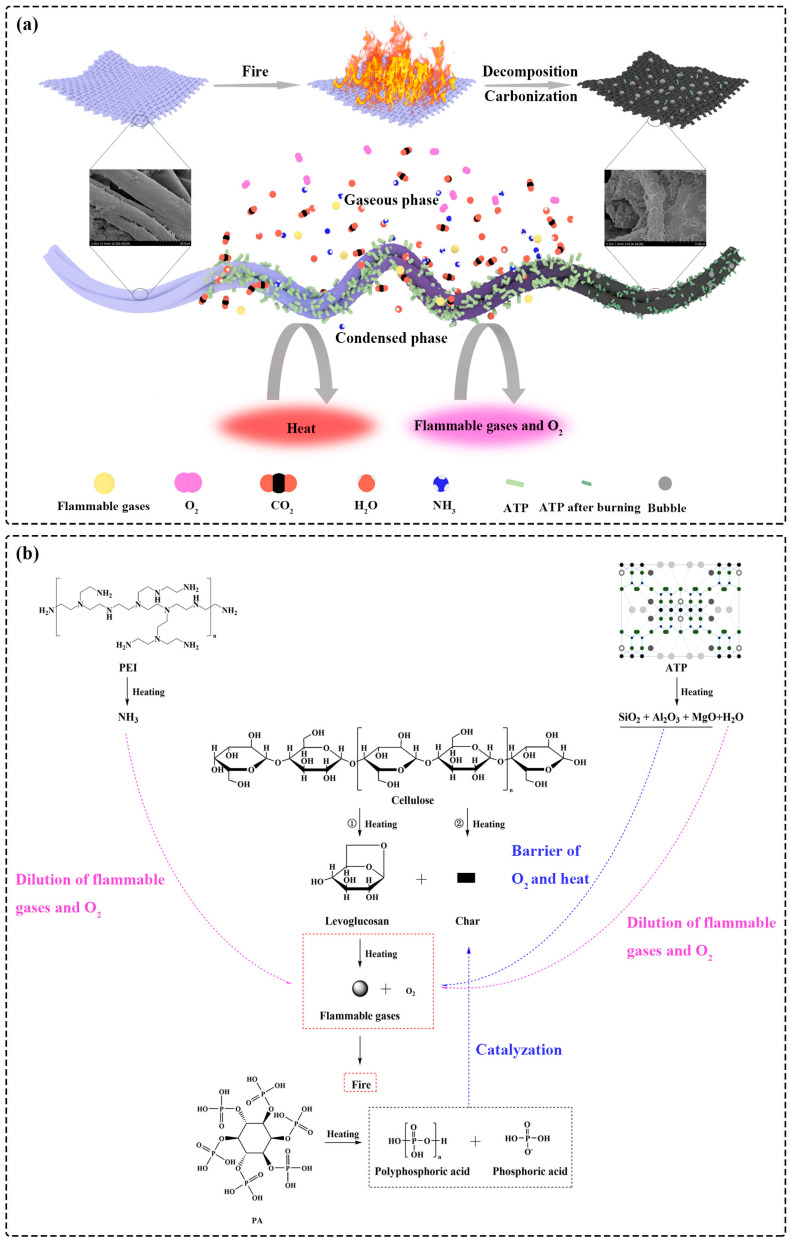
Schematic illustration of the intumescent flame-retardant mechanism during (**a**) the combustion process and (**b**) its specific chemical reaction.

Fuel, O_2_, and heat are three essential elements of combustion, and for whichever element is restricted, the act of burning cannot proceed completely. As described in Figure 7a, the release of the flammable gases which were fuels of combustion decreased in the gaseous phase. Furthermore, the produced inert gases diluted the concentration of the fuel and O_2_ around the combustion area. In the condensed phase, the stable char with clay particles prevented the transfer of fuel and O_2_ between the underlying cotton fibers and the combustion area. The heat was also blocked by the char and clay particles. As a result, the collaborative contribution of the gaseous and condensed phases achieved the flame retardancy of the prepared cotton fabrics.

The effect of the flame retardants in the condensed and gaseous phases is illustrated in Figure 7b. Usually, there were two competitive reactions for cellulose pyrolysis which are the formations of char and levoglucosan. The char layer could protect underlying fibers from further decomposition. However, levoglucosan is of great flammability and can be decomposed into several volatile fuel species. Due to the exposure to constant heat sources, the combustion started with the reaction between flammable gases and O_2_ from the surrounding atmosphere. PA as an acid source was decomposed into polyphosphoric acid and phosphoric acid at a high temperature. The acid species catalyzed the dehydration process and promoted the carbonization of cellulose to produce a carbonaceous layer instead of levoglucosan. As a result, the graphitization degree of the residual char was improved, indicating the formation of the dense isolation char layers. Furthermore, ATP was decomposed into thermally stable substances, including SiO_2,_ MgO, and Al_2_O_3_, which acted as a physical barrier to hinder the transport of heat, O_2_, and flammable volatile organic compounds, achieving flame retardancy in the condensed phase. In the gaseous phase, nonflammable gases were released during the carbonization process, resulting in the swollen char layer with some bubbles. PEI took a part of the blowing agent and produced inert NH_3_ to dilute the concentration of O_2_ and fuels in the air and prevent flame propagation. Besides, ATP could release moisture which further improved flame retardancy in the gaseous phase. Therefore, the flame-retardant effect of the coatings was achieved in the condensed and gaseous phases.

### 3.7. Mechanical Properties of the Control and FR Cotton Fabrics

**Figure 8:**
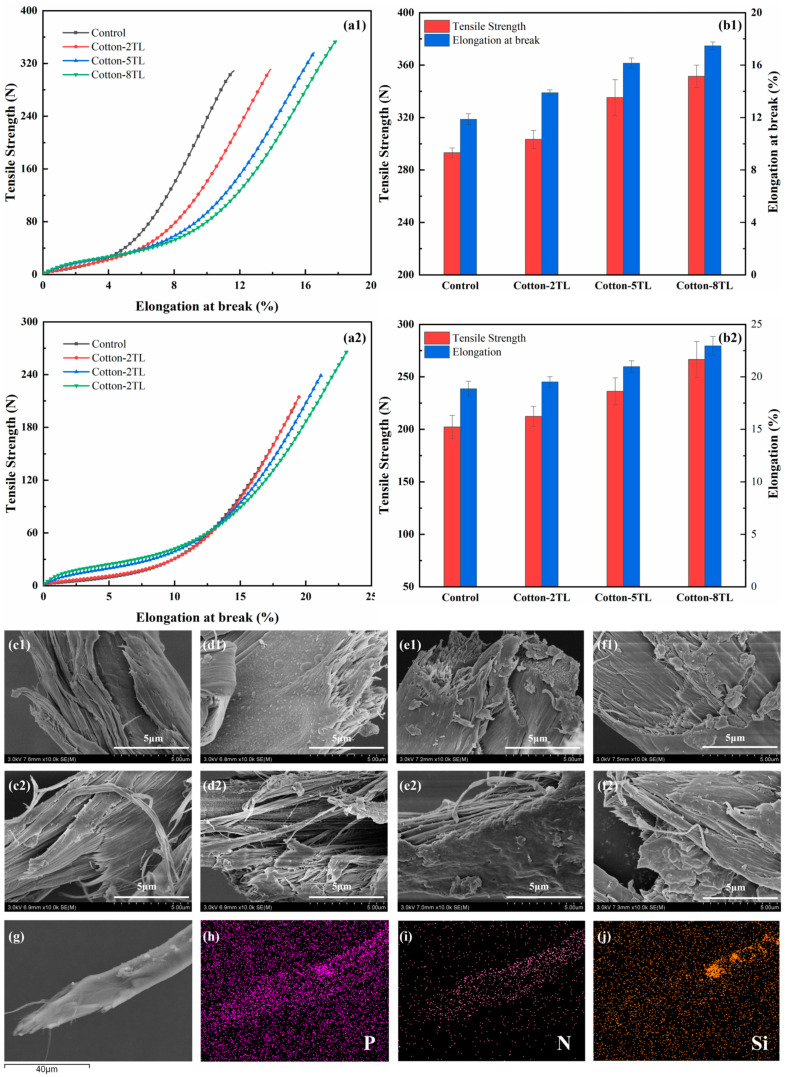
Stress-strain curves and mechanical properties of samples in the (**a1**,**b1**) warp and (**a2**,**b2**) weft direction. SEM images of the fracture area of the (**c1**,**c2**) Control, (**d1**,**d2**) Cotton-2TL, (**e1**,**e2**) Cotton-5TL, (**f1**,**f2**) Cotton-8TL in the warp and weft direction. (**g**–**j**) EDS mapping of element distribution on the fracture area of Cotton-8TL in the warp direction.

The effects of the coatings on the mechanical property of the FR cotton fabrics both in the warp and weft directions were studied. As shown in Figure 8a,b, both the breaking strength and the elongation at break were reinforced whether in the warp or weft direction. The increase in coating layer numbers was advantageous for improving the tensile strength of the FR cotton fabrics. Compared with those of the control cotton fabrics, the breaking strength of Cotton-8TL was increased by 20% and 32% in the warp and weft directions, respectively. In terms of the elongation at break, Cotton-8TL had an increment of 47% in the warp direction and 22% in the weft direction, respectively. As described in Figure 8c–f, the fracture area of the control cotton fibers was smooth, but the surface of fibers after LbL assembly was obviously wrapped with relatively dense coating layers. It is worth noting that P, N, and Si were found inside the fibers (Figure 8g–j, Figures S5 and S6), revealing that a certain amount of PEI, PA, and clay particles infiltrated the interior of the fibers during the LbL assembly. Accordingly, not only the dense coating layers on the surface of the fibers but also the intermolecular forces between the flame retardants and cellulose collaboratively dominated in the enhancement of mechanical properties as shown in Figure 1c. In addition, the instinct laminated chain structure of ATP which was a one-dimensional material could build connections between fibers and might take a role in improving mechanical properties [56]. Not only the flame retardancy but also the mechanical properties of cotton fabrics were reinforced at the same time through a facile LbL assembly method.

## 4. Conclusions

In summary, this work provided an efficient synergistic flame-retardant coating consisting of intumescent and clay-based flame retardants. PEI, ATP, and PA were successfully deposited on the surface of cotton fabrics and endowed cotton fabrics with both effective flame retardancy and improved mechanical properties. Compared with the control cotton fabrics, all the FR cotton fabrics show higher LOI values, and the LOI value of Cotton-8TL was 27.0% which increased by 57%. The PHRR values of the FR cotton fabrics decreased, and the PHRR of Cotton-2TL declined by 41%, indicating the surface coatings show effective flame retardancy. The mechanism of flame retardant could be attributed to the blocking role of the dense char layer in the condensed phase and the dilution effect of flammable gases and O_2_ in the gaseous phase. In addition, the well-constructed flame-retardant coating improved the mechanical properties effectively. The tensile strength at the break of Cotton-8TL increased by 20% in the warp direction and 32% in the weft direction, respectively. Therefore, the fabricated eco-friendly flame-retardant coatings balanced the flame retardancy and mechanical properties of cotton fabrics simultaneously.

## Figures and Tables

**Table 1:** Atomic concentrations of all cotton fabrics measured by XPS.

Samples	C (%)	O (%)	N (%)	P (%)	Si (%)	Mg (%)	Al (%)
Control	71.30	28.70	0.00	0.00	0.00	0.00	0.00
Cotton-2TL	56.23	33.33	5.19	2.50	1.14	0.65	0.96
Cotton-5TL	53.03	29.69	9.00	3.94	1.59	2.28	0.47
Cotton-8TL	52.57	27.47	11.70	3.53	0.81	3.47	0.44

**Table 2:** The related CCT data and LOI values for all samples.

Samples	Add-On (%)	PHRR (kW/m^2^)	THR (MJ/m^2^)	FIGRA (kW/m^2^/s)	LOI (%)
Control	-	265.6	2.63	15.6	17.2
Cotton-2TL	10.5	157.2	2.05	9.8	19.8
Cotton-5TL	28.3	192.2	2.71	12.8	22.8
Cotton-8TL	32.7	197.5	2.89	13.2	27.0

## Data Availability

The data presented in this study are available on request from the corresponding author.

## Supplementary Materials

The following supporting information can be downloaded at: https://www.mdpi.com/article/10.3390/polym14224994/s1, Figure S1: (a) Wide-scan XPS spectra of the control and FR cotton fabrics and (b) Si2p spectra of FR cotton fabrics; Figure S2: Elemental mapping of element distribution on the surface of Cotton-8TL; Figure S3: FTIR spectra of the control and FR cotton fabrics; Figure S4: EDS mapping of element distribution on the surface of the char residue for Cotton-8TL; Figure S5: EDS mapping of element distribution on the fracture surface of (a) Control, (b) Cotton-2TL, (c) Cotton-5TL, (d) Cotton-8TL in the weft direction; Figure S6: EDS mapping of element distribution on the fracture surface of (a) Control, (b) Cotton-2TL, and (c) Cotton-5TL in the warp direction; Table S1: TGA and DTG data of samples under nitrogen atmosphere.

## References

[1] Zhang Y., Tian W., Liu L., Cheng W., Wang W., Liew K.M., Wang B., Hu Y. (2019). Eco-friendly flame retardant and electromagnetic interference shielding cotton fabrics with multi-layered coatings. Chem. Eng. J..

[2] Li N., Han H., Li M., Qiu W., Wang Q., Qi X., He Y., Wang X., Liu L., Yu J. (2021). Eco-friendly and intrinsic nanogels for durable flame retardant and antibacterial properties. Chem. Eng. J..

[3] Han X., Wang J., Wang X., Tian W., Dong Y., Jiang S. (2022). Finite Element Analysis of Strengthening Mechanism of Ultrastrong and Tough Cellulosic Materials. Polymers.

[4] Li T., Chen C., Brozena A.H., Zhu J.Y., Xu L., Driemeier C., Dai J., Rojas O.J., Isogai A., Wagberg L. (2021). Developing fibrillated cellulose as a sustainable technological material. Nature.

[5] Zheng C., Sun Y., Cui Y., Yang W., Lu Z., Shen S., Xia Y., Xiong Z. (2021). Superhydrophobic and flame-retardant alginate fabrics prepared through a one-step dip-coating surface-treatment. Cellulose.

[6] Li P., Wang B., Xu Y.-J., Jiang Z., Dong C., Liu Y., Zhu P. (2019). Ecofriendly flame-retardant cotton fabrics: Preparation, flame retardancy, thermal degradation properties, and mechanism. ACS Sustain. Chem. Eng..

[7] Ambekar R.S., Deshmukh A., Suarez-Villagran M.Y., Das R., Pal V., Dey S., Miller J.H., Machado L.D., Kumbhakar P., Tiwary C.S. (2020). 2D hexagonal boron nitride-coated cotton fabric with self-extinguishing property. ACS Appl. Mater. Interfaces.

[8] Zope I.S., Foo S., Seah D.G.J., Akunuri A.T., Dasari A. (2017). Development and evaluation of a water-based flame retardant spray coating for cotton fabrics. ACS Appl. Mater. Interfaces.

[9] Liu Z., Xu M., Wang Q., Li B. (2017). A novel durable flame retardant cotton fabric produced by surface chemical grafting of phosphorus- and nitrogen-containing compounds. Cellulose.

[10] Zhao P., Xu F., Chen Y., Huang T., Zhang G. (2022). A novel durable flame retardant for cotton fabrics based on diethylenetriamine. Polym. Degrad. Stab..

[11] Li N., Chen P., Liu D., Kang G., Liu L., Xu L., Yu J., Li F., Wu D. (2022). Novel P/Si based nanoparticles for durable flame retardant application on cotton. Cellulose.

[12] Nguyen Thi H., Vu Thi Hong K., Ngo Ha T., Phan D.N. (2020). Application of plasma activation in flame-retardant treatment for cotton fabric. Polymers.

[13] Lin D., Zeng X., Li H., Lai X., Wu T. (2019). One-pot fabrication of superhydrophobic and flame-retardant coatings on cotton fabrics via sol-gel reaction. J. Colloid Interface Sci..

[14] Cheng X.-W., Liang C.-X., Guan J.-P., Yang X.-H., Tang R.-C. (2018). Flame retardant and hydrophobic properties of novel sol-gel derived phytic acid/silica hybrid organic-inorganic coatings for silk fabric. Appl. Surf. Sci..

[15] Zhang D., Williams B.L., Shrestha S.B., Nasir Z., Becher E.M., Lofink B.J., Santos V.H., Patel H., Peng X., Sun L. (2017). Flame retardant and hydrophobic coatings on cotton fabrics via sol-gel and self-assembly techniques. J. Colloid Interface Sci..

[16] Yang J.C., Liao W., Deng S.B., Cao Z.J., Wang Y.Z. (2016). Flame retardation of cellulose-rich fabrics via a simplified layer-by-layer assembly. Carbohydr. Polym..

[17] Pan H., Wang W., Pan Y., Song L., Hu Y., Liew K.M. (2015). Formation of self-extinguishing flame retardant biobased coating on cotton fabrics via Layer-by-Layer assembly of chitin derivatives. Carbohydr. Polym..

[18] Shi X.-H., Liu Q.-Y., Li X.-L., Du A.-K., Niu J.-W., Li Y.-M., Li Z., Wang M., Wang D.-Y. (2022). Construction phosphorus/nitrogen-containing flame-retardant and hydrophobic coating toward cotton fabric via layer-by-layer assembly. Polym. Degrad. Stab..

[19] Qiu X., Li Z., Li X., Zhang Z. (2018). Flame retardant coatings prepared using layer by layer assembly: A review. Chem. Eng. J..

[20] Zhang X., Xu Y., Zhang X., Wu H., Shen J., Chen R., Xiong Y., Li J., Guo S. (2019). Progress on the layer-by-layer assembly of multilayered polymer composites: Strategy, structural control and applications. Prog. Polym. Sci..

[21] Lazar S.T., Kolibaba T.J., Grunlan J.C. (2020). Flame-retardant surface treatments. Nat. Rev. Mater..

[22] Xia W., Fan S., Xu T. (2021). Inhibitory action of halogen-free fire retardants on combustion and volatile emission of bituminous components. Sci. Prog..

[23] Jiang Z., Li H., He Y., Liu Y., Dong C., Zhu P. (2019). Flame retardancy and thermal behavior of cotton fabrics based on a novel phosphorus-containing siloxane. Appl. Surf. Sci..

[24] Zhou T., Xu H., Cai L., Wang J. (2020). Construction of anti-flame network structures in cotton fabrics with pentaerythritol phosphate urea salt and nano SiO_2_. Appl. Surf. Sci..

[25] Chen H., Zhu S., Zhou R., Wu X., Zhang W., Han X., Wang J. (2022). Thermal Degradation Behavior of Thiol-ene Composites Loaded with a Novel Silicone Flame Retardant. Polymers.

[26] Wang J., Wu X., Zhou R., Han W., Han X. (2022). Effect of a Novel Graphene on the Flame Retardancy and Thermal Degradation Behavior of Epoxy Resin. J. Macromol. Sci. Part B.

[27] El-Shafei A., ElShemy M., Abou-Okeil A. (2015). Eco-friendly finishing agent for cotton fabrics to improve flame retardant and antibacterial properties. Carbohydr. Polym..

[28] Zhang Z., Ma Z., Leng Q., Wang Y. (2019). Eco-friendly flame retardant coating deposited on cotton fabrics from bio-based chitosan, phytic acid and divalent metal ions. Int. J. Biol. Macromol..

[29] Chen H.-Q., Xu Y.-J., Jiang Z.-M., Jin X., Liu Y., Zhang L., Zhang C.-J., Yan C. (2019). The thermal degradation property and flame-retardant mechanism of coated knitted cotton fabric with chitosan and APP by LBL assembly. J. Therm. Anal. Calorim..

[30] Alongi J., Carletto R.A., Di Blasio A., Carosio F., Bosco F., Malucelli G. (2013). DNA: A novel, green, natural flame retardant and suppressant for cotton. J. Mater. Chem. A.

[31] Alongi J., Di Blasio A., Milnes J., Malucelli G., Bourbigot S., Kandola B., Camino G. (2015). Thermal degradation of DNA, an all-in-one natural intumescent flame retardant. Polym. Degrad. Stab..

[32] Carosio F., Di Blasio A., Alongi J., Malucelli G. (2013). Green DNA-based flame retardant coatings assembled through layer by layer. Polymer.

[33] Zilke O., Plohl D., Opwis K., Mayer-Gall T., Gutmann J.S. (2020). A flame-retardant phytic-acid-based LbL-coating for cotton using polyvinylamine. Polymers.

[34] Barbalini M., Bartoli M., Tagliaferro A., Malucelli G. (2020). Phytic acid and biochar: An effective all bio-sourced flame retardant formulation for cotton fabrics. Polymers.

[35] Li Y., Wang B., Sui X., Xie R., Xu H., Zhang L., Zhong Y., Mao Z. (2018). Durable flame retardant and antibacterial finishing on cotton fabrics with cyclotriphosphazene/polydopamine/silver nanoparticles hybrid coatings. Appl. Surf. Sci..

[36] Carosio F., Fontaine G., Alongi J., Bourbigot S. (2015). Starch-based layer by layer assembly: Efficient and sustainable approach to cotton fire protection. ACS Appl. Mater. Interfaces.

[37] Xing C.Y., Zeng S.L., Qi S.K., Jiang M.J., Xu L., Chen L., Zhang S., Li B.J. (2020). Poly (vinyl alcohol)/beta-cyclodextrin composite fiber with good flame retardant and super-smoke suppression properties. Polymers.

[38] Alongi J., Poskovic M., Visakh P.N., Frache A., Malucelli G. (2012). Cyclodextrin nanosponges as novel green flame retardants for PP, LLDPE and PA6. Carbohydr. Polym..

[39] Zhang D., Zeng J., Liu W., Qiu X., Qian Y., Zhang H., Yang Y., Liu M., Yang D. (2021). Pristine lignin as a flame retardant in flexible PU foam. Green Chem..

[40] Kulkarni S., Xia Z., Yu S., Kiratitanavit W., Morgan A.B., Kumar J., Mosurkal R., Nagarajan R. (2021). Bio-based flame-retardant coatings based on the synergistic combination of tannic acid and phytic acid for nylon-cotton blends. ACS Appl. Mater. Interfaces.

[41] Zhang A.-N., Zhao H.-B., Cheng J.-B., Li M.-E., Li S.-L., Cao M., Wang Y.-Z. (2021). Construction of durable eco-friendly biomass-based flame-retardant coating for cotton fabrics. Chem. Eng. J..

[42] Wang S., Tan L., Xu T. (2022). Synergistic effects of developed composite flame retardant on VOCs constituents of heated asphalt and carbonized layer compositions. J. Clean. Prod..

[43] Chen S., Li X., Li Y., Sun J. (2015). Intumescent flame-retardant and self-healing superhydrophobic coatings on cotton fabric. ACS Nano.

[44] Li Y.C., Schulz J., Mannen S., Delhom C., Condon B., Chang S., Zammarano M., Grunlan J.C. (2010). Flame retardant behavior of polyelectrolyte-clay thin film assemblies on cotton fabric. ACS Nano.

[45] Patra A., Kjellin S., Larsson A.-C. (2020). Phytic acid-based flame retardants for cotton. Green Mater..

[46] Liu Y., Liu S., Yin C. (2014). Synthesis and structure-property of polyamide 6/macrogol/attapulgite nanocomposites. Polym. Compos..

[47] Bao Y., Li X., Tang P., Liu C., Zhang W., Ma J. (2019). Attapulgite modified cotton fabric and its flame retardancy. Cellulose.

[48] Gao D., Zhang Y., Lyu B., Wang P., Ma J. (2019). Nanocomposite based on poly(acrylic acid)/attapulgite towards flame retardant of cotton fabrics. Carbohydr. Polym..

[49] Xie H., Lai X., Wang Y., Li H., Zeng X. (2019). A green approach to fabricating nacre-inspired nanocoating for super-efficiently fire-safe polymers via one-step self-assembly. J. Hazard. Mater..

[50] Wang B., Xu Y.-J., Li P., Zhang F.-Q., Liu Y., Zhu P. (2020). Flame-retardant polyester/cotton blend with phosphorus/nitrogen/silicon-containing nano-coating by layer-by-layer assembly. Appl. Surf. Sci..

[51] Apaydin K., Laachachi A., Ball V., Jimenez M., Bourbigot S., Toniazzo V., Ruch D. (2013). Polyallylamine–montmorillonite as super flame retardant coating assemblies by layer-by layer deposition on polyamide. Polym. Degrad. Stab..

[52] Xin S., Yang H., Chen Y., Yang M., Chen L., Wang X., Chen H. (2015). Chemical structure evolution of char during the pyrolysis of cellulose. J. Anal. Appl. Pyrolysis.

[53] Rao W., Shi J., Yu C., Zhao H.-B., Wang Y.-Z. (2021). Highly efficient, transparent, and environment-friendly flame-retardant coating for cotton fabric. Chem. Eng. J..

[54] Liu L., Pan Y., Zhao Y., Cai W., Gui Z., Hu Y., Wang X. (2020). Self-assembly of phosphonate-metal complex for superhydrophobic and durable flame-retardant polyester–cotton fabrics. Cellulose.

[55] Guo W., Wang X., Huang J., Zhou Y., Cai W., Wang J., Song L., Hu Y. (2020). Construction of durable flame-retardant and robust superhydrophobic coatings on cotton fabrics for water-oil separation application. Chem. Eng. J..

[56] Huang D., Wang W., Xu J., Wang A. (2012). Mechanical and water resistance properties of chitosan/poly(vinyl alcohol) films reinforced with attapulgite dispersed by high-pressure homogenization. Chem. Eng. J..

