# Supplementary material for: Fabrication of an Eco-Friendly Clay-Based Coating for Enhancing Flame Retardant and Mechanical Properties of Cotton Fabrics via LbL Assembly
Source: Polymers (Basel). 2022 Nov 18;14(22):4994. doi: 10.3390/polym14224994 (PMC9695412; doi:10.3390/polym14224994)
Supplement: Supplementary file 1 [file polymers-14-04994-s001.zip › polymers-1999970-supplementary.pdf]

Supporting Information

Fabrication of an eco-friendly clay-based coating for enhancing flame retardant and mechanical properties of cotton fabrics via LbL assembly

Mingjia Kang, Silu Chen *, Rongjie Yang *, Dinghua Li and Wenchao Zhang

National Engineering Research Center of Flame Retardant Materials, School of Materials Science and Engineering, Beijing Institute of Technology, Beijing 100081, China

* Correspondence: chensilu@bit.edu.cn (S.C.); yrj@bit.edu.cn (R.Y.)

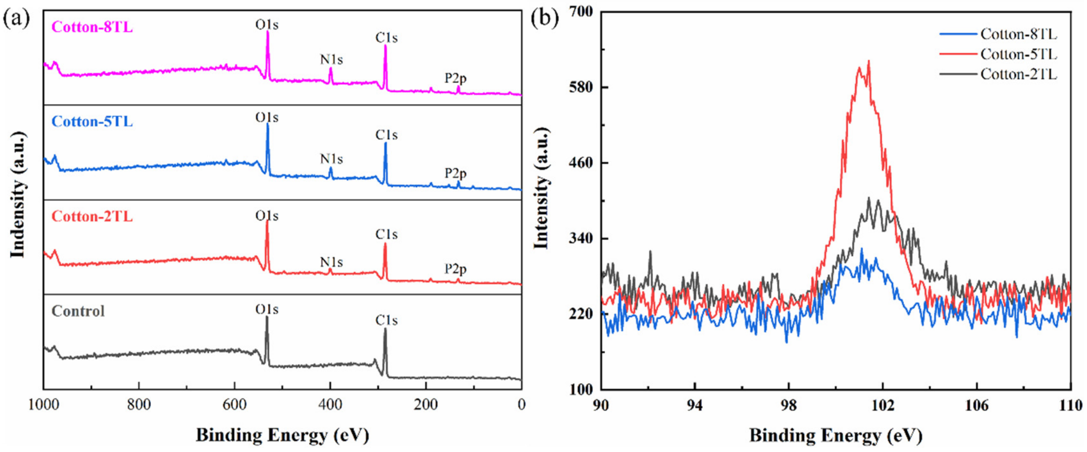

Figure S1. (a) Wide-scan XPS spectra of the control and FR cotton fabrics and **(b)** Si_{2p} spectra of FR cotton fabrics.

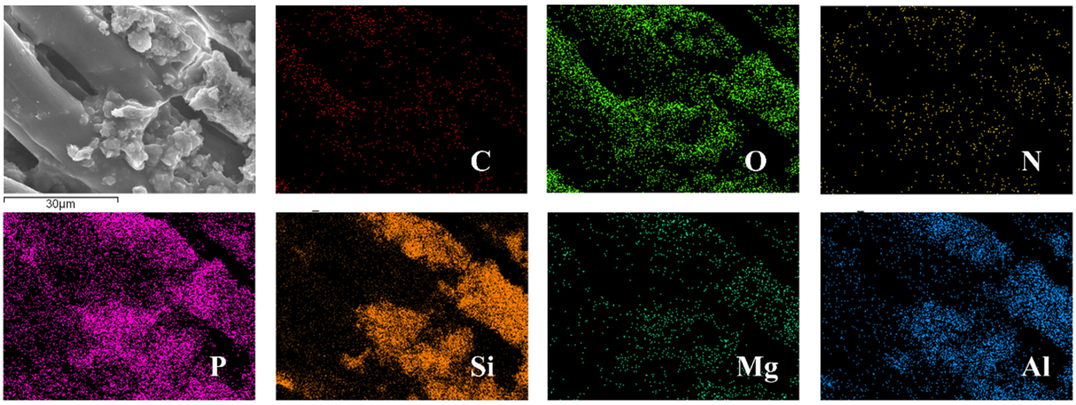

Figure S2. Elemental mapping of element distribution on the surface of Cotton -8TL.

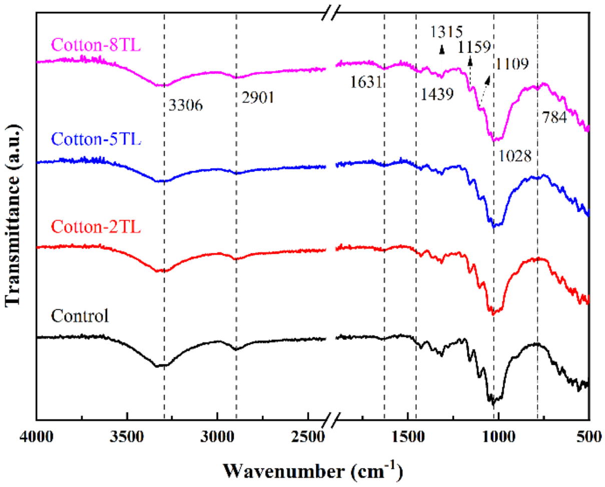

Figure S3. FTIR spectra of the control and FR cotton fabrics.

Table S1. TGA and DTG data of samples under nitrogen atmosphere.

Samples	$T_{-5\%}$ (°C)	T_{max} (°C)	Residue at 700°C (wt%)
Control	323.3	367.8	12.4
Cotton-2TL	295.2	316.0	32.3
Cotton-5TL	306.5	321.3	41.0
Cotton-8TL	268.5	325.0	43.9

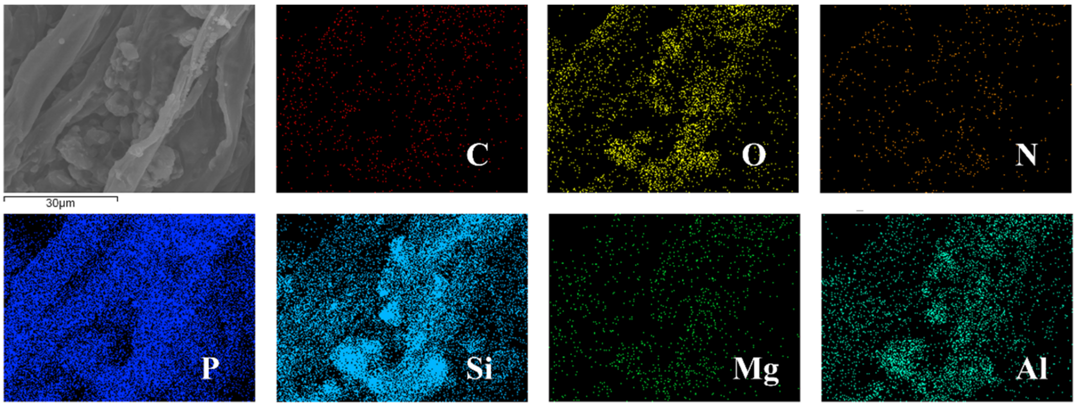

Figure S4. EDS mapping of element distribution on the surface of the char residue for Cotton-8TL.

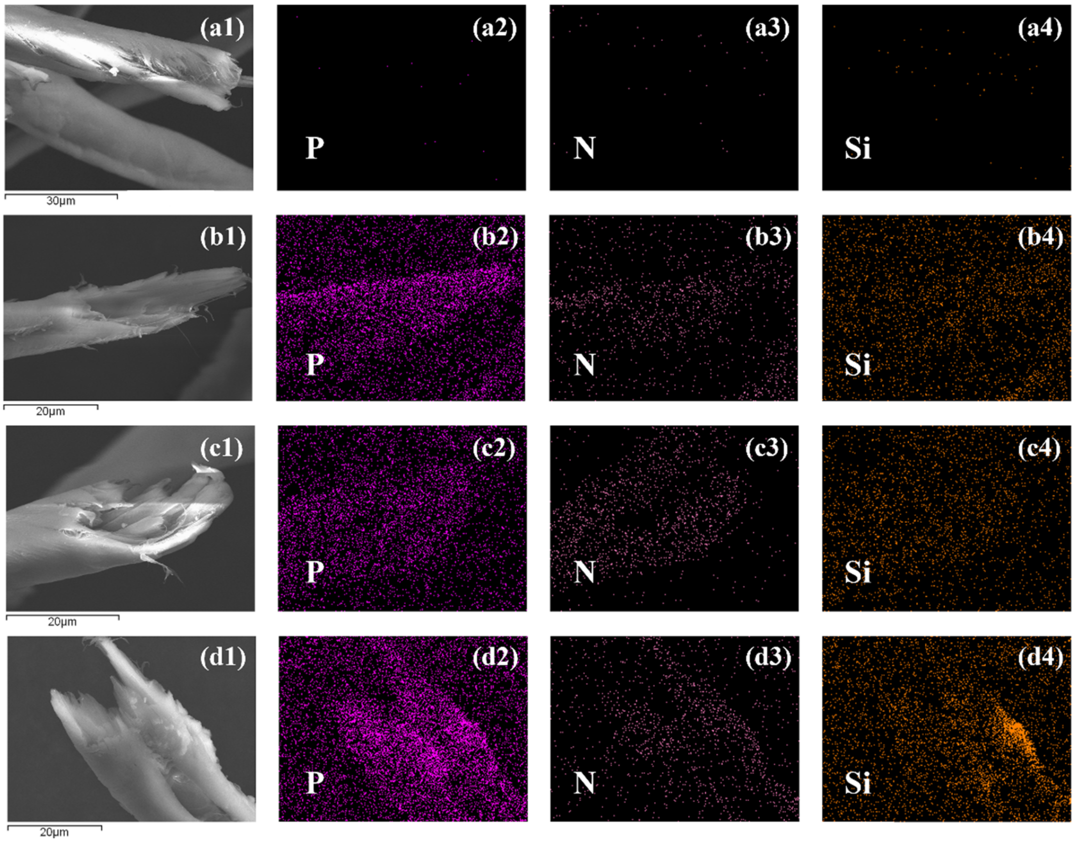

Figure S5. EDS mapping of element distribution on the fracture surface of **(a)** Control, **(b)** Cotton-2TL, **(c)** Cotton-5TL, **(d)** Cotton-8TL in the weft direction.

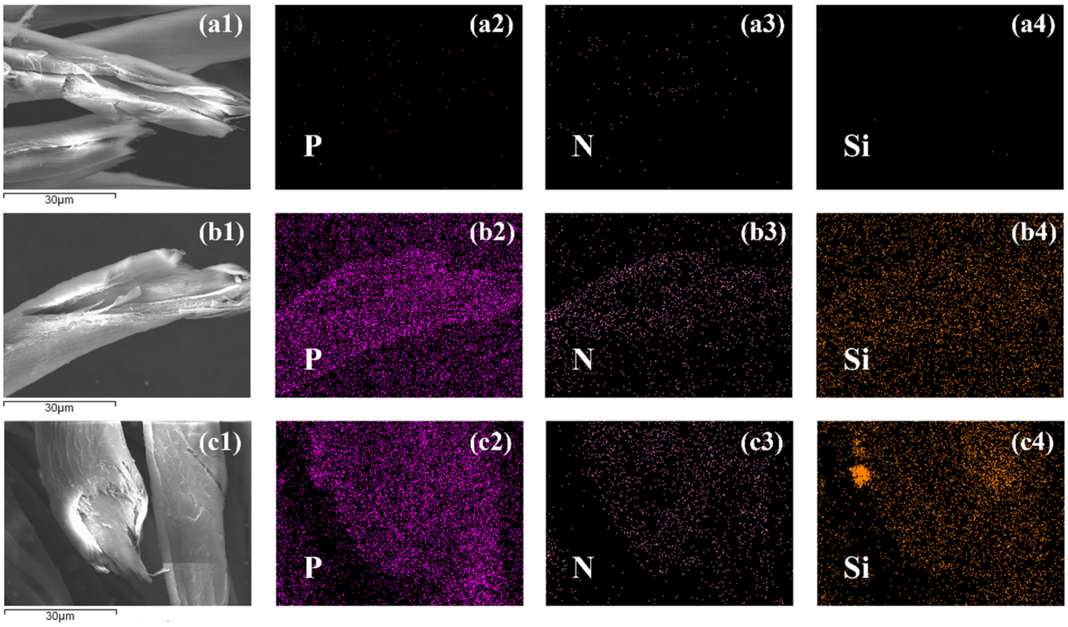

Figure S6. EDS mapping of element distribution on the fracture surface of **(a)** Control, **(b)** Cotton-2TL, and **(c)** Cotton-5TL in the warp direction.
